# To be or not be (in the LAD): emerging roles of lamin proteins in transcriptional regulation

**DOI:** 10.1042/BST20210858

**Published:** 2022-04-19

**Authors:** Ezequiel Nazer

**Affiliations:** Universidad de Buenos Aires (UBA), Facultad de Ciencias Exactas y Naturales, Departamento de Fisiología, Biología Molecular y Celular and CONICET-UBA, Instituto de Fisiología, Biología Molecular y Neurociencias (IFIBYNE), Buenos Aires, Argentina

**Keywords:** chromatin topology, laminA, lamina-associated-domains (LADs), laminB, nuclear lamina, topologically associated-domains (TADs)

## Abstract

Lamins are components of the nuclear lamina, a protein meshwork that underlies the nuclear membrane. Lamins interact with chromatin in transcriptionally silent regions defined as lamina-associated-domains (LADs). However, recent studies have shown that lamins regulate active transcription inside LADs. In addition, ChIP-seq analysis has shown that lamins interact with lamin-dependent promoters and enhancers located in the interior of the nucleus. Moreover, functional studies suggest that lamins regulate transcription at associated-promoters and long-range chromatin interactions of key developmental gene programs. This review will discuss emerging, non-canonical functions of lamins in controlling non-silent genes located both inside and outside of LADs, focusing on transcriptional regulation and chromatin organization in *Drosophila* and mammals as metazoan model organisms.

## Introduction

**Figure 1.**
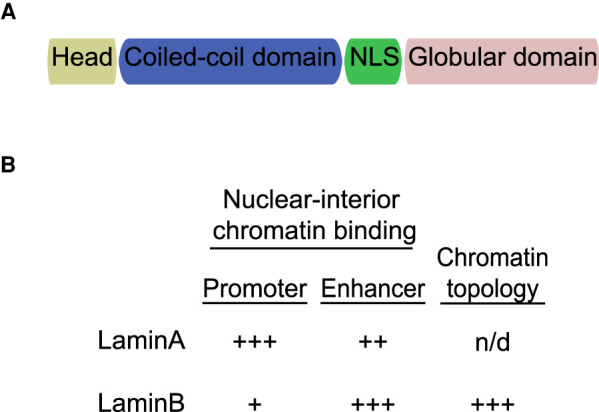
Canonical structure and functional roles of lamins. (**A**) Lamins have three domains: a head domain, a coiled-coil rod domain that mediates interactions with other lamina proteins, a nuclear localization signal (NLS), and an Ig-like fold globular domain that mediates interactions with non-lamina proteins. (**B**) Association of lamins with promoters and enhancers located in the interior of the nucleus and their impact in chromatin topology is represented with plus symbols ranging from lower (+) to higher (+++) enrichment/effect [21,42,49,50,52,53,57].

An intriguing question in biology is how do cells differentiate and maintain their status over successive rounds of cell division. During development, gene expression is tightly regulated to execute specific gene programs. Among the different layers of regulation, spatial genome organization has emerged as a key mechanism [1,2]. The nuclear envelope (NE) plays a critical role in integrating extracellular signals and modulating gene expression regulation. The NE is formed by the inner and outer nuclear membranes and the nuclear pore complex (NPC). In addition, nuclear lamins which correspond to filamentous type V proteins form a protein meshwork at the periphery of the nucleus known as the nuclear lamina (NL) which covers the inner face of the NE [3]. Lamins are restricted to metazoans and categorized into two types, A and B, based on their biochemical and sequence characteristics. Almost all invertebrates have B-type lamins, with some exceptions, such as *Drosophila*, which has one B-type (*lamin*) and one A-type lamin (*lamin C*) gene, respectively [4]. The majority of vertebrates have one A-type lamin and two B-type lamin genes. In mammalian cells, A-type lamins are produced by alternative splicing, resulting in the major isoforms LaminA and LaminC (hereafter both referred to as LaminA) [5]. The two B-type proteins, Lamin B1 and Lamin B2 (hereafter LaminB), are encoded by two separate genes *lamin B1* and *lamin B2* [6,7]. A-type lamins are expressed in differentiated cells but minimally detectable in pluripotent stem cells and during early embryogenesis. In contrast, B-type lamins are expressed in all somatic cell types [8–10]. The biological significance of the cell-type specificity and species specificity for different subtypes remains unknown. Lamin proteins are structurally characterized by a central α-helical rod domain containing heptad repeats surrounded by a ∼30 amino acid non-helical amino terminal domain (head) and a highly conserved immunoglobulin-like fold (Ig fold) C-terminal domain (tail) [11]. In addition, lamins contain a classical SV-40-type nuclear localization sequence that is located between the central alpha-helical rod and Ig fold domains (Figure 1). In concordance with intermediate filament proteins, lamins self-assemble into more complex structures forming coiled-coil dimers, which additionally arrange themselves in a head-to-tail orientation leading to protofilament formation.

Functionally, lamins have been implicated in a large repertoire of cellular mechanisms including mechanotransduction, modulation of nuclear stiffness and elasticity, chromatin organization, DNA replication and repair, and transcriptional regulation [12]. Pioneering electron microscopy-based studies show close proximity of condensed chromatin and the NL, indicating that patches of heterochromatin can be located in the nuclear periphery [13]. These findings were reinforced by DNA fluorescence *in situ* hybridization (FISH) experiments that confirmed that certain transcriptionally silent regions associated with heterochromatin are located in the nuclear periphery interacting with NL. More recently, genome-wide assays based on DNA adenine methyltransferase identification (DamID) and ChIP-seq, mostly performed to profile LaminB, identified genomic regions that frequently make close contact with the NL. These domains referred to as lamina-associated domains (LADs) are characterized by showing low transcriptional activity and heterochromatin features [14–17].

Spatial partitioning of the genome is also evident in interaction maps generated by chromosome conformation capture technologies such as Hi-C [18,19]. Genome-interaction maps have revealed that highly self-interacting genomic units define functional and structural domains known as topologically associated domain (TADs) [20]. The genomic regions within TADs have higher frequency of 3D interaction with each other relative to sequences located in other TADs. In addition, chromatin can also be organized in compartments [20]. These structures correspond to the largest scale of chromosome organization and are defined as sets of spatial proximal TADs. In addition, compartments comprise TADs that show long-range interactions at a statistically higher observed frequency than expected, but it is also worth noting that the actual inter-TAD interaction frequencies are very low.

Functionally, compartments can be transcriptionally sub-classified as A-type (open) or B-type (closed) depending on their transcriptionally active or inactive status, respectively. DamID and Hi-C data from mouse embryonic fibroblasts (MEFs) demonstrated that LaminB LADs correspond to the B-compartment [21]. Importantly, LADs are extremely conserved between species, although some show a certain degree of dynamism. In fact, it has been recently reported that a significant fraction of genes are able to escape transcriptional repression despite embedding in LAD–chromatin, thus supporting that LADs are dynamic structures capable of modulating active transcription [22]. This piece of evidence suggests that lamins are likely engaged in different layers of gene expression regulation, ranging from transcription to genome organization. In fact, mutations in genes encoding nuclear lamins induce transcriptional changes associated with a large repertoire of human diseases, such as ageing, fibrosis and cancer [23–27]. It is evident that proper spatial positioning of the genome is important for development and health; however, the precise molecular mechanisms by which lamins regulate gene expression requires deeper investigation. This review covers the emerging non-canonical functions of lamins in controlling non-silent genes located inside and outside LADs, focusing on transcription regulation and chromatin organization in *D. melanogaster* and mammals as metazoan model organisms.

## Lamina-associated domain organization

LADs were first mapped by DNA adenine methyltransferase (DamID) identification (DamID) in *Drosophila* and later by ChIP-seq and tyramide signal amplification (TSA)-sequencing (TSA-seq) mostly using LaminB as target protein [17,28–31]. In brief, DamID is based on fusing the protein of interest to a DNA adenine methyltransferase that methylates proximal DNA, which is afterwards isolated for deep-sequencing. LADs are relatively larger patches of mainly heterochromatin corresponding to histone repressive marks such as H3K9me2/3 and to a lesser extent H3K27me3 [14,15,17]. In mammals, cells have ∼1000–1500 LADs with a typical size between 10 kb to 10 Mb (with a median of ∼0.5 Mb). LADs also exist in *Drosophila* with similar characteristics to the human genome but 5-fold smaller, consistent with its much more compact genome [17,28,32].

LADs can also be classified according to their epigenetic compositions and cell type-specific genome interactions. Across species and cell types, LADs are categorized into regions that associate with the NL in all cell types (constitutive, cLADs) and regions that facultatively associate with the NL in a cell type-specific manner (facultative, fLADs) [15,16,33]. cLADs are enriched for H3K9me2/3 histone marks, whereas fLADs are enriched for H3K27me3. A noteworthy feature of fLADs is that they contain genes that are developmentally regulated. In this regard, several efforts have been made to understand the link between chromatin status, gene expression and gene localization at the nuclear periphery in *Drosophila* and mammalian cells.

The majority of the aforementioned studies have shown that genes located at the nuclear periphery are transcriptionally repressed, whereas detachment from LADs is correlated with gene activation [34–36]. Moreover, random integration of reporter genes to the nuclear periphery frequently leads to their transcriptional repression [37]. Conversely, ectopic relocalization of lamin-associated genes into the interior of the nucleus can induce transcriptional activation [37]. However, it has been recently shown that chromatin decondensation induced with synthetic transcription factors promotes repositioning of peripheral genes towards the nuclear interior decoupled from transcription activation in mammals [38].

In *Drosophila*, it has been shown that during neuroblast differentiation the transcription factor Hunchback is susceptible to be transcribed during a competence window. During that time window, the *hunchback* gene (*hb*) is repressed by transcriptional repressors before its movement to the nuclear periphery where it becomes permanently silenced [35]. Importantly, LaminB depletion reduces repositioning of the *hb* gene to the nuclear periphery, extends the transcriptional window competence and decreases *hb* transcriptional silencing. Overall, these results suggest that lamins play a role as primary hierarchical corepressor by maintaining the silenced state, at least for the *hb* locus.

It has been shown that ∼10% of genes are able to escape transcriptional repression despite being located along LADs [37]. In this regard, single-cell analysis using a DamID derivative approach that allows tracking of the fate of LADs over time showed that NL contact frequency (CF) varies across the genome, where a significant proportion of LADs show high cell-to-cell variability in NL associations [33]. Importantly, loci embedded within regions that interact less consistently with the NL, might exhibit more cell-to cell transcription plasticity. Indeed, the proportion of genes with active transcription negatively correlates with CF, thus implying that the CF of LADs within a population of cells directly relates to gene transcriptional escape [33]. Interestingly, LAD tracking during mitosis showed that many LADs that were in contact with the NL in the mother cell (‘mother LADs') are then located in the nuclear interior in the daughter cells [39]. These observations suggest that peripheral positioning of LADs after mitosis is a stochastic event that might contribute to the transcriptional leakage of a proportion of LAD genes and reinforces the notion that ‘escaper' genes are not necessarily associated with the NL.

## Relationship between lamins and active transcription

Historically, it has been proposed that genes embedded within the NL are necessarily repressed. This paradigm has been mostly based on single locus scale analysis. However, the advent of genome-wide studies has challenged this consensus. Strikingly, recent experiments showed that lamins are able to modulate active transcription inside LADs. For instance, global run-on sequencing (GRO-seq) experiments showed that a significant fraction of promoters located inside LADs are actively transcribed in mammals [22,40]. These ‘escaper' promoters are located within LADs but locally detached from the NL and contain relatively low levels of H3K9me2/3, which could facilitate their transcription. Interestingly, ‘escaper' genes show lower nascent RNA and RNA Pol II occupancy in gene bodies relative to promoters. Moreover, these observations are also in agreement with the relative lower levels of H3K36me3, a mark of elongation, in ‘escaper' gene bodies. These chromatin features suggest that ‘escaper' promoters are in a paused state that might be released under certain physiological conditions.

Recently, a comprehensive study using TALE-VP16 and CRISPR tethering approaches to activate LAD genes showed that NL detachment takes place along the entire gene body, which is more pronounced near the promoter region compared with the downstream transcription unit in mouse embryonic stem (ES) cells [41]. Interestingly, such NL dissociation can be extended to several hundred kb including neighboring non-transcribed genes that still maintain their silent status. In addition, the degree of NL detachment is inversely correlated with the transcriptional level of the genes tested. In this line, the authors showed compelling evidence suggesting a role for transcription elongation to counteract NL interactions [41]. A plausible explanation could be attributed to a physical effect where the elongating RNA polymerase complex displaces NL-interacting chromatin proteins, thus increasing the detachment from the NL. However, further experiments will be required to understand the actual molecular mechanism.

In agreement with mammalian studies, genome-wide transcriptional analyses by nascent euRNA-seq (neuRNA-seq) in *Drosophila* Kc167 cells (Kc) showed that LaminB depletion enhances transcription in domains known as RED chromatin located within LADs [42]. In brief, this chromatin feature has been characterized in *Drosophila* and corresponds to transcriptionally active regions characterized by the lack of the elongation histone mark H3K36me3. In addition, RED chromatin is associated with developmentally regulated genes or those susceptible to induction by external stimuli [43]. Interestingly, genes whose promoters are in RED chromatin that are also inside a LAD are still expressed under normal conditions but at lower levels than those in RED chromatin outside of LADs. In this line, it has been shown that RED chromatin is connected to RNA Pol II pausing, a process implicated in transcription modulation, controlled mainly by the interplay between the negative elongation factor (NELF-E) and the elongating-related factor CDK9 [44,45]. In this vein, it has been previously shown that LaminB forms a nuclear complex with RNA Pol II in flies [42]. Overall, these findings in *Drosophila* reinforce the interplay between lamins and RNA Pol II to modulate transcription inside LADs likely by controlling transcriptional pausing, as has been suggested in mammals (see above).

Nuclear lamins have also been observed in the interior of the nucleus [46,47]. The nuclear-interior lamins are soluble, mobile, and unpolymerized. In addition, depolymerization of nuclear lamins is regulated by phosphorylation of specific serine residues during the cell cycle and upon changes in the mechanical environment in interphase cells [8,48]. These observations have raised the tantalizing possibility that lamins may modulate gene expression by binding genomic sites outside LADs. However, the molecular link to support such a possibility has remained elusive, mainly because the pool of lamins associated with euchromatin has been refractory to detection by DamID [49,50]. In agreement with the hypothesis of functional nuclear-interior lamins, microarray and RNA-seq analysis showed transcriptional changes outside LADs in LaminB knockout embryonic stem cells and trophectoderm cells in mice [51]. In addition, GRO-seq analysis from human LaminA knockout skin fibroblast cells showed transcriptional effects mainly outside LADs [52,53]. Strikingly, immunofluorescence microscopy coupled with ChIP-seq analysis showed that the depolymerized form of LaminA phosphorylated at Ser22 (pS22-LaminA) is: (1) highly abundant in the nuclear interior and (2) enriched at discrete sites of accessible chromatin associated with active promoters and enhancers outside LADs (Figure 1). With respect to its molecular role, integration of LaminA ChIP-seq profiles with GRO-seq analysis in LaminA KO cells showed that nuclear-interior pS22-LaminA modulates the transcriptional cycle of active promoters and enhancers outside LADs [52,53]. Moreover, the absence of LaminA leads to an accumulation of RNA Pol II along gene bodies and an increase in transcriptional elongation, likely by affecting RNA Pol II pause release. Together, these results suggest that depolymerized lamins have gene regulatory functions outside the NL. Finally, given that a significant proportion of lamin-affected genes are located outside LADs, it is tempting to speculate that lamins also modulate transcription by controlling distal regulatory elements [21,42,54]. However, it is also plausible that transcriptional misregulation relies on downstream effects following from disruption of the lamin-associated genes.

## ChIP-seq and DamID lamin profile differences

As mentioned above, LADs have initially been discovered by DamID assays. In addition, large chromatin domains with similar properties have been identified by chromatin immunoprecipitation (ChIP) coupled to high-throughput sequencing (ChIP-seq). The datasets reveal significant overlap in genomic coverage of lamin-interacting domains discovered by DamID and ChIP. However, a recurrent topic of debate in the field relies on the technical challenges to isolate and profile nuclear-interior lamins. As discussed above, recent ChIP-seq genome-wide studies have shown that lamins contact euchromatin in the interior of the nucleus [50,54–56]. In this regard, a critical step for ChIP-seq experiments corresponds to the sonication step. It has been suggested that sonication resistance differences between euchromatin and heterochromatin might contribute to enriching the former since moderate shearing (100–600 bp) favors enrichment for euchromatin [50,55,56]. On the other hand, heterochromatin yields larger fragments (>1 kb) that may be discarded or difficult to sequence. Conversely, higher sonication enriches heterochromatin whereas euchromatin is lost as a result of hyperfragmention. Therefore, the extent of chromatin shearing for the ChIP protocol influences the detection of lamin interacting chromatin domains by ChIP-seq. Alternatively, the antibody of choice may greatly affect lamin profiling [50]. For instance, LaminA ChIP-seq profiling using the reference anti-pan-N-terminal-Lamin A/C (aa2–29) monoclonal antibody that recognizes both the phospho-S22 and non-phospho-S22 forms exhibited LAD localization, whereas pS22-LaminA ChIP-seq showed enrichment outside of LADs [52]. Therefore, the location and accessibility of lamin epitopes can significantly affect ChIP results.

One of the most important differences between the two approaches is that ChIP provides a snapshot of protein occupancy at a single point in time. On the other hand, DamID relies on DNA methylation that occurs over a time window of several hours. DamID shows chromatin-binding events that occur *in vivo*, in contrast with ChIP, which assays interactions after cell crosslinking. Finally, it should be noted that DamID has been extensively used to profile transcription factors in euchromatin [43]. However, the localization of LaminB in different landscapes, such as euchromatin and heterochromatin, might explain its difficulty to be detected in euchromatin genome-wide. Indeed, it has been shown that the interaction between chromatin and LaminB is more stable at the NL [43,49]. Perhaps, DamID favors the identification of stable interactions between lamins and chromatin that are likely to occur at the nuclear periphery. Alternatively, since the DamID assay relies on Dam-fusion proteins, it might be possible that the Dam-Lamin fusion protein functions normally at the nuclear periphery, but is impaired to form functional complexes in the nuclear interior.

## Role of lamins in 3D genome topology

In addition to *cis* regulatory elements, transcription is modulated by distal enhancers which provide complex and precise control of gene expression through long-range interactions. As mentioned above, nuclear-interior pS22-LaminA binds sites with chromatin features of active enhancers in human skin fibroblasts [53]. Importantly, gain of pS22-LaminA is associated with increased presence of c-Jun transcription factor and H3K27ac levels at active enhancers in progeria-patient fibroblasts [53]. Moreover, pS22-LaminA binding is increased at enhancers linked with a subset of abnormally activated genes that are highly relevant to progeria phenotypes [53]. Together, these results provide molecular evidence that nuclear-interior pS22-LaminA is associated with progeria disease by increasing enhancer activity and inducing the gene-program associated with such disease [53]. However, the molecular interplay between LaminA and associated-enhancers with target promoters requires further investigation.

In contrast with A-type, B-type lamins have been shown to remain tightly associated with the nuclear membrane. However, recent LaminB ChIP-seq analysis on euchromatin enriched fractions showed a significant association between LaminB binding and transcription start sites (TSSs) that do not overlap with canonical LADs in mouse mammary gland cells [49]. Importantly, the specificity of the antibody used for the genome-wide mapping of LaminB was confirmed at certain loci by LaminB immunoprecipitation from LaminB-depleted cells; however, it would be interesting to perform LaminB ChIP-seq in LaminB knockout cell lines to confirm its association with TSSs outside LADs genome-wide. Further characterization showed that LaminB binds discrete, actively expressed, open euchromatin sites clustered in regions of ∼0.3 Mb that the authors termed euchromatin LADs (eLADs) to differentiate them from conventional LADs. Importantly, LaminB enrichment levels are positively correlated with the transcriptional activity of LaminB-bound around TSS. In addition, eLADs change dynamically during epithelial-to-mesenchymal (EMT) transformation, an essential process during generation of tissues and organs and also implicated in cancer development. ChIP-seq integrated with GRO-seq analysis showed that 50% of genes differentially transcribed upon LaminB depletion are direct targets of LaminB outside LADs [49]. Importantly, a significant proportion of these genes are related, but not restricted, to the EMT transcriptional program, thus implying a more general role of LaminB in gene regulation. In addition, LaminB was found to be related with genomic spatial architecture during EMT transformation by modulating the configuration of topologically associated domains (TADs). TADs are partitioned by architectural proteins that define their borders which contribute to impair ectopic interactions with regulatory elements [1]. It has been shown that LaminB enrichment at TAD borders is enhanced upon EMT differentiation, establishing more stable borders and increased intra-TAD interactions that may fine-tune enhancer–promoter interactions and subsequently transcription during EMT [49].

An intriguing question of the field relies in understanding the interdependence between spatial genome organization and the establishment of chromatin topology. In this regard, it has been recently shown that LADs are undetectable in oocytes but become *de novo* established after fertilization in mouse embryos [57]. Interestingly, LAD profiling during development combined with HiC data showed that LADs are defined in zygotes before TAD establishment. In contrast with TADs, A and B compartments are detected in early zygotic stages and the majority of LADs overlap with B compartments. Unexpectedly, a significant fraction of LADs are present in A compartments in two-cell embryos. A deeper LAD dynamic analysis showed that *de novo* LAD establishment in the two-cell stage that remains as LADs during embryo development occurs before B compartment formation, thus raising the interesting possibility that for certain genomic regions LADs may contribute to prime the formation of B compartments [57]. Finally, it is worth mentioning that it has been shown that lamins are dispensable for overall LAD organization and transcriptional regulation in murine embryonic stem cells [54]. This is not surprising as many proteins of the inner nuclear membrane bind to chromatin [36,58–62]. In this regard, it should be noted that murine embryonic stem cells are characterized by a rapid cell cycle, a more dynamic NL architecture and less stable incorporation of LaminB, which might have evolved to alternative mechanisms based on non-lamin proteins to drive LAD formation.

**Figure 2.**
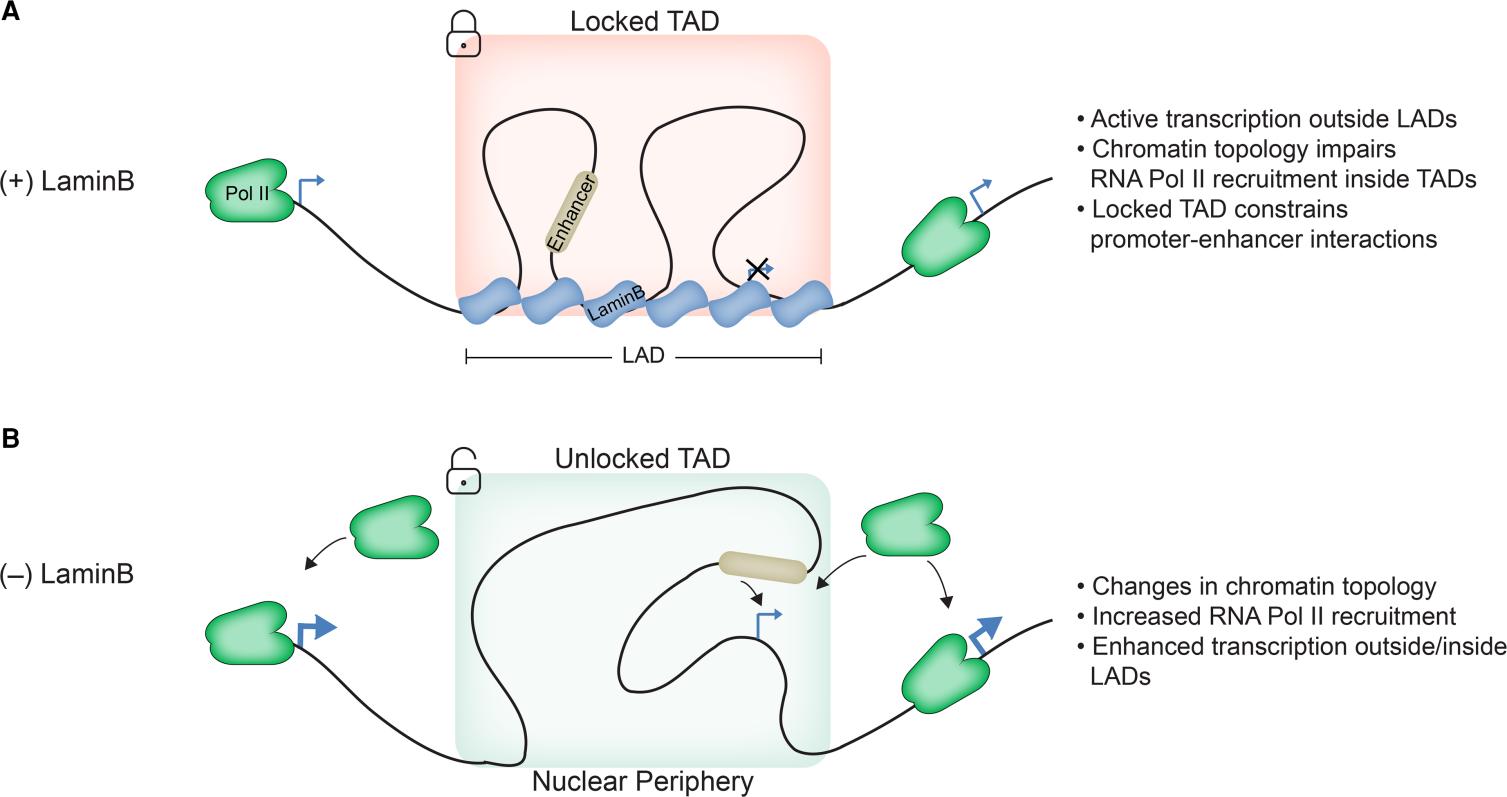
Role of LaminB in 3D organization and its impact on transcription regulation. (**A**) LaminB associates with LAD/TAD. LaminB scaffolding helps maintain the TAD in a locked structure, likely impairing RNA Pol II recruitment and preventing promoter–enhancer interactions across the TAD. (**B**) Upon depletion of LaminB the chromatin topology from the LAD/TAD switches from a locked to a more accessible unlocked structure, thus permitting inter-and-intra-TAD interactions. As a result, RNA Pol II recruitment and promoter–enhancer interaction may be increased.

In concordance with the role of lamins in transcription and chromatin organization described in mammals, it has been shown that certain spermatogenesis gene clusters are associated with the nuclear periphery and become de-repressed and reposition toward the nuclear interior upon depletion of LaminB in *Drosophila* somatic cells [34]. Interestingly, the *nht* gene, encoding a transcription factor implicated in the spermatogenesis gene program, is also inactive and embedded within LADs [42]. However, *nht* becomes up-regulated upon LaminB depletion without being repositioned towards the interior of the nucleus. Chromosome conformation capture assays showed that LaminB controls the overall chromatin topology of the LAD and TAD in which *nht* is located [42]. Specifically, interactions between *nht* and other sites within the TAD decrease in interaction frequency in LaminB-depleted cells. In addition, increased interactions are observed between the *nht* promoter and sequences located in other chromatin domains beyond 1 Mb away. These large alterations in topology in LaminB-depleted cells could explain the resultant transcriptional increase in *nht* by allowing interaction with inappropriate enhancers or otherwise creating a more permissive transcriptional environment [42] (Figure 2). These results are in concordance with recent findings in mice in which the absence of lamins reduces the frequency of inter-TAD interactions associated with constitutive LADs and simultaneously enhances inter-TAD interactions between TADs embedded within LADs and TADs located in the interior of the nucleus [21] (Figure 1). Moreover, higher enhancer activity is associated with increased interactions with active chromatin and transcriptional up-regulation. On the other hand, loss of active enhancers correlated with more frequent interactions with repressive chromatin domains and decreased transcriptional output.

Deeper analysis of LaminB DamID profiles from MEFs under normal growth conditions showed discrete 1–25 kb regions of low interactions with the NL known as disruption in peripheral signal (DiP) [21]. Intriguingly, the majority of DiPs are enriched for active enhancers that interact with the active A-compartment and show preferential interaction with promoters located outside LADs, thus suggesting that these regions loop away from the NL [21]. In agreement with these findings, more recent LaminB ChIP-seq analysis of undifferentiated mouse embryonic stem cells (mESCs) showed similar breaks in large contiguous domains at the nuclear periphery. These domains, termed H3K9me2-only domains (KODs), are enriched in H3K9me2 (a specific mark of nuclear peripheral chromatin) but feature minimal LaminB association [63]. Unlike DiPs, each KOD can span tens to hundreds of kilobases (KOD median domain size: 70 kb, average domain size: 159 kb). In addition, KODs are depleted of active enhancers but enriched in ‘poised' and tissue-specific enhancers that can become activated upon mESC differentiation. Strikingly, it has been recently reported that certain enhancers in humans, but not their dependent promoters, gain and lose LAD occupancy in a cell type-specific manner, thus reinforcing the concept that LADs are dynamic domains implicated in cell type specification by ‘priming' and ‘poisoning' regulatory elements during cell differentiation [64]. Together, these findings support that lamins organize 3D chromatin interaction networks at several layers including: active/inactive TADs, enhancer activity and promoter interactions, which all subsequently modulate transcription inside and outside LADs during cell differentiation. Altogether, substantial progress has been made in uncovering how lamins modulate transcription, especially for those located outside LADs. With this expanding knowledge of lamin gene regulatory functions, it will be important to delve further into the potential interplay between lamins, the transcriptional machinery and additional architectural proteins to better understand how lamins fine-tune proper chromatin interactions and transcription.

## Summary

The classical view in the nuclear organization field assumes that lamins are restricted to LADs. These domains have been characterized to interact with heterochromatin regions located at the nuclear periphery. Initially, the interplay between lamins and chromatin has been analyzed by *in situ* hybridization and genome-wide DamID profiles [14,15,17,65]. On the other hand, microscopy studies have also shown that lamins can be detected into the interior of the nucleus in different organisms [55,66,67]. However, this paradigm has been challenged since cumulative results show that lamins are able to control transcription outside LADs, thus suggesting a functional role in euchromatin. This is also consistent with recent LaminA and LaminB ChIP-seq analysis showing that the vast majority of their binding sites are associated with active promoters and enhancers genome-wide in human skin fibroblasts and mouse mammary epithelial cells, respectively [49,52]. Importantly, a large proportion of LaminA-bound promoters are transcriptionally affected in LaminA KO predominantly outside LADs. However, the molecular link to understand those results remains elusive, mainly because the lamin population enriched in euchromatin has been refractory to being detected by DamID [49,50].

Currently, it is unclear how lamins regulate transcription at associated TSSs. GRO-seq analysis suggested that LaminA restricts transcriptional elongation likely by promoting pausing [52]. In addition, lamins associate with distal regulatory enhancers and help to organize 3D chromatin topology. In this regard, it is likely that lamins organize proper long-range interactions between enhancers and target promoters to modulate the activity of pausing and/or elongation factors and fine-tune transcription. In agreement with this hypothesis, *Drosophila* LaminB modulates active transcription associated with RED chromatin, a hallmark associated with pausing and stimulus-responsive transcription, and chromatin topology [42]. In this scenario, it is tempting to speculate that lamins might work as dynamic cofactors that control elongating RNA Pol II, thus allowing developmental and tissue-restricted genes to remain in an activatable state when gene activity is desired upon cell differentiation. However, the emerging non-canonical functions for lamins raise several intriguing questions that still need to be addressed.

## Perspectives

- Lamins have been traditionally seen as components of the NE associated with heterochromatin and transcriptional silencing.
- Recent genome-wide approaches show that lamins are associated with promoters and enhancers outside LADs and regulate transcription and chromatin topology of key differentiation gene programs.
- Further experiments will shed light on understanding how lamins regulate promoters and enhancers and their therapeutic potential as novel cofactors to treat diseases such as ageing and cancer.

## Abbreviations

CF: contact frequency
EMT: epithelial-to-mesenchymal transformation
LADs: lamina-associated-domains
MEFs: mouse embryonic fibroblasts
NE: nuclear envelope
NL: nuclear lamina
TADs: topologically associated domain
TSSs: transcription start sites
